# Remote and at-home data collection: Considerations for the NIH HEALthy Brain and Cognitive Development (HBCD) study

**DOI:** 10.1016/j.dcn.2022.101059

**Published:** 2022-01-06

**Authors:** Sean C.L. Deoni, Viren D’Sa, Alexandra Volpe, Jennifer Beauchemin, Julie M. Croff, Amy J. Elliott, Nicolò Pini, Maristella Lucchini, William P. Fifer

**Affiliations:** a Maternal, Newborn, and Child Health Discovery & Tools, Bill & Melinda Gates Foundation, Seattle, WA, USA; b Advanced Baby Imaging Lab, Rhode Island Hospital, Providence, RI, USA; c Department of Pediatrics, Warren Alpert Medical School at Brown University, Providence, RI, USA; d Center for Health Sciences, National Center for Wellness and Recovery, Oklahoma State University, Tulsa, USA; e Avera Research Institute, Sioux Falls, SD, USA; f Department of Psychiatry and Pediatrics, Columbia University Medical Center, New York State Psychiatric Institute, 1051 Riverside Drive, New York, NY 10032, USA

**Keywords:** Remote data collection, Mobile MRI, Neurodevelopment, Environmental exposure, Child development, Personal technology

## Abstract

The NIH HEALthy Brain and Cognitive Development (HBCD) study aims to characterize the impact of *in utero* exposure to substances, and related environmental exposures on child neurodevelopment and health outcomes. A key focus of HBCD is opioid exposure, which has disproportionately affected rural areas. While most opioid use and neonatal abstinence syndrome has been reported outside of large cities, rural communities are often under-represented in large-scale clinical research studies that involve neuroimaging, in-person assessments, or bio-specimen collections. Thus, there exists a likely mismatch between the communities that are the focus of HBCD and those that can participate. Even geographically proximal participants, however, are likely to bias towards higher socioeconomic status given the anticipated study burden and visit frequency. Wearables, ‘nearables’, and other consumer biosensors, however, are increasingly capable of collecting continuous physiologic and environmental exposure data, facilitating remote assessment. We review the potential of these technologies for remote in situ data collection, and the ability to engage rural, affected communities. While not necessarily a replacement, these technologies offer a compelling complement to traditional ‘gold standard’ lab-based methods, with significant potential to expand the study’s reach and importance.

## 1 Introduction

The HEALthy Brain and Cognitive Development (HBCD) Study is a planned prospective and longitudinal study of brain and cognitive development across the first 10 years of life (Volkow et al., 2020). The focus of the study is to characterize healthy normative neurobehavioral development, as well as potential aberrant development resulting from, or associated with, *in utero* substance (opioids and other) use and in the context of pre- and post-natal environmental exposures. The HBCD study will also provide novel insight on the neurodevelopmental impact of the COVID-19 pandemic and associated economic shut-down, social-distancing, and other public health policies that have fundamentally altered the economic, social, and psychosocial environments experienced by families and children.

As a study of neurodevelopment, a combination of multimodal neuroimaging techniques, namely advanced 3 T magnetic resonance imaging (MRI) and electroencephalography (EEG) is planned to thoroughly characterize maturing brain structure and function. In addition, evolving child cognition and behavior will be assessed across all major domains, including early development and pre-academic skills, intellectual functioning, expressive and receptive language, visuomotor/visuospatial functioning, fine motor coordination, and attention and executive functioning. Additional maternal and child health information will provide context to this detailed neuroimaging and psychometric data, and will include anthropometry, physiology, sleep, physical activity, family history, caregiver interaction and bonding; biospecimens (genetics, microbiome, breastmilk, blood, hair, shed teeth, urine, and toenails); and demographic and socioeconomic indicators (race, ethnicity, parent education and family income, and residential and neighborhood characteristics). These elements will provide rich and important characterization of pre- and post-natal health and associated exposure influences previously shown to shape child health and developmental outcomes.

**Fig. 1:**
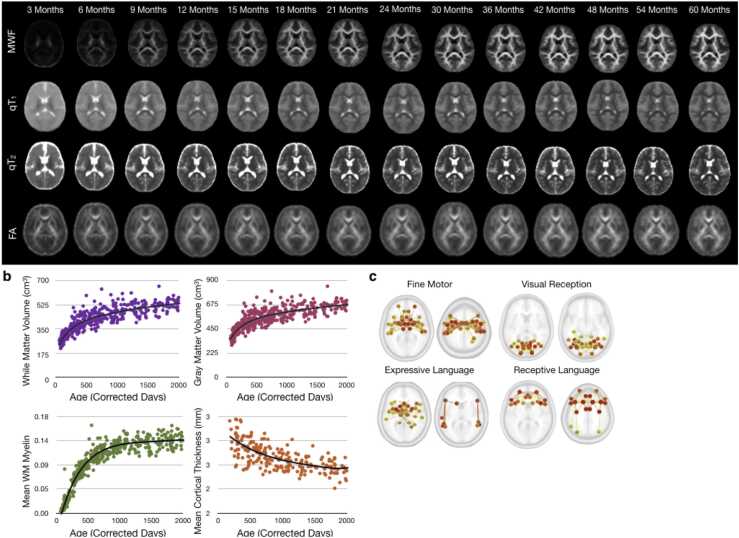
(a) General multimodal MRI patterns of brain maturation, including indices of anatomy, myelination, and functional connectivity across the first 5 years of life. (b) Curated and condensed trajectories of white and gray matter volume, myelination, and mean cortical thickness showing the changing rate of development, with most developmental processes and changes occurring rapidly over the first 1000 days and the slowing throughout the remainder of childhood. (c) Networks of functional connectivity change associated with emerging fine motor, language, and visual processing skill.

The developmental interval spanning infancy to pre-adolescence is widely recognized as an important and sensitive period of health and development. Across this age-span, the structural and functional architecture of the brain is established, and life-long patterns of mental and physical (cardiovascular and metabolic) health are set in motion. From birth to age 10, the human brain increases more than four-fold in volume (Houston et al., 2014), and its eloquent neuroarchitecture matures through processes including neurogenesis and migration, synaptogenesis and pruning, and cortical and white matter myelination (Fig. 1). These structural and functional changes support the emergence and refinement of nearly every cognitive, behavioral, and academic skill (Clouchoux et al., 2012, Lyall et al., 2015, Makropoulos et al., 2016, Gao et al., 2017). This remarkable transformation is fueled by the mother and child’s nutritional and energy resources, and shaped by cascades of genetic and environmental interactions that are modulated through psychosocial and caregiving relationships (Rice and Barone, 2000, Stiles and Jernigan, 2010, Liu et al., 2012, Fields, 2015, Choi et al., 2019). While neurodevelopmental changes occur across the lifespan, the period before age 10 represents a period of peak growth, maximal plasticity, and unique sensitivity (Gale et al., 2004).

The rapid neurodevelopmental growth and achievement of major developmental milestones across the first decade of life demands careful consideration of the study visit timing and frequency necessary to accurately characterize neurodevelopment. The near exponential growth over the first 1000 days of development (e.g., Fig. 1), for example, may suggest at least biannual imaging assessments; whereas the slower growth exhibited throughout older childhood may be adequately captured with only annual visits. However, even a frequent visit schedule may not capture the specialized microstructural or functional changes that likely accompany achievement of major developmental milestones (e.g., rolling over, first steps, first words) or life-events (e.g., introduction to formal schooling, changes in living conditions, and initial onset of puberty). While most past neuroimaging studies of development have utilized a standardized set of imaging timepoints (e.g., 6, 12, and 24 months), a potentially more informative approach would be base scanning timepoints on the developmental progress of the child (e.g., when they first roll over or take their first steps). Such frequent neuroimaging, however, would be exceptionally challenging, particularly for participants not proximal to the research setting. Whether performed during the day or in the evening, infant scanning can require prolonged and repeated study visits for the child to fall asleep and to collect all desired data elements. This can become more challenging during the toddler and early childhood periods (2–5 years) when children sleep less throughout the day but are difficult to routinely scan awake (Richardson et al., 2018).

While children are inherently competent in their ability to initiate relationships, explore, seek meaning, and learn; they are vulnerable and depend entirely on caregivers for their survival, emotional security, modeling of behaviors, and the nature and rules of the physical and socio-cultural world that they inhabit. The infant brain is likewise born with immense capacity to learn, remodel, and adapt, but sensitive and vulnerable to neglect and environmental exposures that begin even before birth (Fulford et al., 2003, Kisilevsky et al., 2009, Bock et al., 2015, Lynema et al., 2016, Vohr et al., 2017). Optimal brain development, therefore, depends on secure and trusting relationships with knowledgeable caregivers who are sensitive and responsive to the infant’s needs and interests (Garnett et al., 2020). Myelination and synaptogenesis, for example, are stimulated by external cues and experiences like maternal interaction, and physical skin-to-skin “kangaroo” care, touch, and warmth (Kolb, 2009, He et al., 2010, Fields, 2015, Ismail et al., 2017, Choi et al., 2019). The brain’s adaptive plasticity, however, is a double-edged sword. While positive and enriching environments can promote healthy brain development (Dobbing, 1964, Bradley and Corwyn, 2002, Noble et al., 2005, Farah et al., 2006, Georgieff, 2007, Swain et al., 2007, Hillman et al., 2008, Hackman and Farah, 2009), neglect, abuse and other negative environments can impair maturing brain systems and lead to disrupted cognitive and behavioral outcomes (Feinberg, 1982; Rees and Inder, 2005; Fields, 2008). Negative environments often, but not always, accompany substance use and exposure that, as may be expected, is also associated with worsened cognitive development and outcomes. Substance-using parents are often less responsive to an infant’s needs nor attentive to their cues for support and interaction (Mozeika et al., 2020).

To appreciate the impact of positive and negative psychosocial and environmental exposures on child neurodevelopment, and the compounding effects of substance exposure, will require extensive and often invasive assessments of the home environment, psychosocial factors, and health metrics. Other pre-and post-natal exposures often associated with opioid use which are relevant to fetal and infant development include maternal sleep health impacted by factors such as depression, drinking and smoking, and low socioeconomic status, and sleep disturbances. These factors have been shown to induce physiological adaptations that may predispose offspring to altered development and disease across the lifespan. While many lab-based assessments of these factors are well developed and accepted, at-home or in situ assessment may be preferable and less sensitive to the Hawthorne effect (i.e., changes in behavior resulting from known observation) (McCambridge et al., 2014). Similarly, 1–3-day nutrition or sleep diaries, or measures of child health concurrent with the imaging visit may not be representative of the 3, 6, or even 12-month period between study visits. The breadth of possible factors that likely impact infant, child, and adolescent development, and the shifting influence with child age, places unique demands on the HBCD study and study participants.

Adding further to the challenges of HBCD is the potential mismatch between the families most able to participate in the study, and the families most important to the study. Opioid use, abuse, overdose, and newborn neonatal abstinence syndrome (NAS) in the US have disproportionally affected rural areas (Becker et al., 2018). Over 90% of opioid users live outside of large cities and city centers, and nearly half of all newborns with NAS are born in rural areas (Kozhimannil et al., 2019). Amongst the hardest hit areas in the US includes rural southwest Virginia, in the heart of Appalachia with an NAS rate of up to 80 per 1000 live births (compared to a national average of 7.3 per 1000). With respect to COVID-19, the changing economic and social landscape has disproportionately affected racial and ethnic minorities, and lower income families (Raifman and Raifman, 2020). Many have been forced to take on additional employment in essential industries to maintain housing, food, and other necessities leaving less time for supportive and interactive parenting (or assisting with home schooling older children). This added economic and health uncertainty has led to increased mental health concerns, higher rates of domestic and inter-partner violence, and escalating substance use (Mukhtar, 2020) - all further exacerbated by the temporary closure of support services.

The overlap between COVID-19 and the opioid epidemic has, tragically, created a perfect storm that has amplified many factors that negatively affect fetal, infant, and child neurodevelopment. However, many of those most affected are the most unable to participate in research studies. Many areas throughout Appalachia, for example, are more than 4 h away from the nearest research center; lower income families may not be able to devote time away from work, school, or family to attend prolonged study visits; and racial and ethnic minorities have often been neglected and maligned by the scientific community and are under-represented in research studies.

To encourage participation amongst remote or rural communities many groups have customized vans and recreational vehicles (RVs) into mobile laboratories that can facilitate traditional lab-based in-person visits but at remote community centers, schools, or even participant homes (Racine and Kobinger, 2019). Mobile labs can provide space for cognitive and behavioral assessments, facilities and storage for biospecimen collections (blood samples, etc.), as well as carry sensitive neuroimaging technology, such as EEG or NIRS (Lau-Zhu et al., 2019).

Beyond mobile labs, the emergence of low cost and innovative technologies has immense potential to address the increasing challenges of subject participation and visit burden, the opportunity to reach under-represented communities, as well as increase the amount of data that can be collected. Consumer wearables, such as Fitbit, Apple Watch, Oura ring, Movesense, Gabi Baby Band and other fitness trackers and wearables can provide continuous and non-invasive physiological monitoring and associated health data, such as activity levels, heart-rate (HR), HR variability (HRV), and sleep metrics. These may be further linked to ‘nearable’ devices related to anthropometry and body composition, heart, and brain activity (ECG and EEG), air quality, light exposure, cardiovascular and pulmonary health (lung function, max VO2), substance use (e.g., breath analysis of blood alcohol content and/or exhaled carbon monoxide), and parent-child language interaction. Connected smart phones and tablets have increasingly been used to conduct remote study visits, including neurocognitive assessments, and remote data capture (online forms and questionnaires, nutrition and food frequency diaries). Research groups have become increasingly proficient at remote biospecimen collection, including saliva, fecal, hair, toe/fingernails, whole blood, and dried blood spots. Combined, these devices make possible near-continuous data collection and monitoring while potentially reducing participant burden. The real-time nature of these devices allows collection of data before, during, and after sensitive developmental periods (potentially not known a priori) without suffering problems associated with retrospective recall.

Remote data collection, via mobile labs, personalized wearable or nearable technologies, or their combination, has appeal to the HBCD study, specifically with respect to increasing participation amongst geographically remote or otherwise under-represented communities; facilitating more frequent data collection that aligns with developmental timelines or milestones; as well as paradoxically reducing subject burden. In this review, we highlight potential approaches that speak to these points, with focus on domains of relevance to HBCD, including neurocognitive assessment, biospecimen collection, health and environmental monitoring, and neuroimaging. Taking a human-centered design (HCD) prospective, which takes the perspective of the participating family, mother, and child, rather than that of the research team, we review potential approaches that could replace and/or complement traditional in-person lab-based assessments, examining challenges with participant convenience, harmonization, and data security. We also explore additional aspects, including research team burden.

## 2 Methods

### 2.1 Neuroimaging

HBCD is fundamentally an imaging study of brain and cognitive development (Volkow et al., 2020). Imaging measures related to developing structure, function, and brain connectivity will be collected using high field (3 Tesla, T) state-of-the-art MRI systems with multi-channel radiofrequency (RF) coil arrays (48 channels or greater) and current 80 mT/m and 200 mT/m/s gradients. These data will be complemented in younger infants and children with electrophysiology (EEG) measures of task-based neurofunction that cannot be easily performed with MRI.

While there is little doubt that 3 T MRI systems provide structural and functional data with high spatial and temporal resolution and fidelity, these systems carry a substantial infrastructure cost. State-of-the-art systems require dedicated imaging suites, substantial uninterrupted power, advanced cooling systems, and experienced and trained technicians to run and support them. As such, they are predominately limited to tertiary care settings and leading university research centers, skewing the individuals and populations that can participate in neuroimaging studies towards proximal and higher socioeconomic demographics. The considerable acquisition and operational cost of these systems means there is often limited time availability - a challenge for infant and child studies where participants cannot be guaranteed to fall asleep and be scanned within the usual 1-hour scan block.

At its most basic level, therefore, the HBCD study is a challenge for HCD as it elevates the imaging equipment at the design center, with all other considerations, including the participating family, secondary.

### 2.2 Portable MRI

**Fig. 2:**
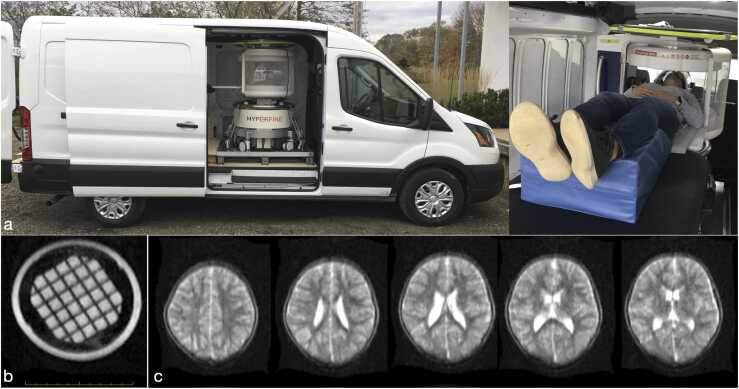
Mobile low field MRI using the 64mT Hyperfine system installed in a customized cargo van (a). Despite its low field and portable nature, the device provides sound imaging abilities without geometric distortions (b) and with image quality and contrast nearing 1.5 T quality (c).

Rather than require participants and families to travel to the imaging center, HCD principles would seek ways to bring the imaging center to the family. The desire for more accessible point-of-care and ‘anywhere/everywhere’ MRI has led to the development of lower field MRI systems (<0.5 T). Using advanced machine learning approaches for denoising and image reconstruction, these systems now offer structural imaging quality nearly on-par with some older generation 1.5 T systems. The light weight (~1400 lbs) Hyperfine Swoop™ (www.hyperfine.io) is one of the first commercially available low field systems. The Swoop has a permanent 64mT main magnetic field, a 5 Gauss boundary diameter of ~5 feet, and low power requirements. Other research-focused systems, with main permanent magnets up to ~100mT have similarly been proposed and demonstrated *in viv*o (Marques et al., 2019, Sarracanie and Salameh, 2020). Without need for costly permanent siting or cryogens, their relatively low weight and low power needs make systems like Hyperfine ideal candidates for mobile imaging, e.g., Fig. 2 with a customized van that can travel on rural secondary, local, and dirt roads without a commercial driver’s license. A more fully equipped lab, with assessment space could also be built out of an RV or motorhome.

The Hyperfine system is currently capable of whole-brain T_1_ and T_2_-weighted structural imaging (Fig. 2), fluid-attenuated contrast, and diffusion imaging, with spatial resolutions approaching 1.5 × 1.5 × 1.5 mm isotropic. Quantitative relaxometry, magnetization transfer, and perfusion and functional imaging methods are currently under investigation. Image quality transfer algorithms(Alexander et al., 2014; Alexander et al., 2017) may offer the potential to significantly improve apparent spatial resolution and image quality, and novel imaging approaches, such as field cycling (Koenig et al., 1975), and hyperpolarized imaging (Coffey et al., 2013) may allow us to explore physiologic and metabolic processes not possible at higher field strengths(Springer et al., 2014; Bai et al., 2018). In combination with either high- or low-density EEG systems, a full complement of structural and functional neuroimaging measures can be collected.

In the context of HBCD and HCD, portable MRI is compelling. The most obvious advantage is the ability to perform routine structural imaging at a participant’s home, wherever they live, at a fraction of the cost and burden of conventional 3 T imaging. We envisage two complementary use-cases: 1. Inclusion of underserved communities and individuals; and 2. Increasing neuroimaging frequency.

For participants in rural areas, the ability to travel two or more hours to the nearest research center or hospital setting is a barrier to participation. Even providing transportation and hotel accommodation reimbursement may not be enough to offset potential economic loss from taking two or more days off work; and may not be feasible if a large or single-parent family needs to travel. In these cases, bringing the lab and scanner to the participant eliminates this barrier. Whilst many imaging centers routinely charge $800 or more per 1-hour scan block, the Hyperfine system may be as low as $50. Given constraints on grant resources, this costing difference could be used to either vastly increase the number of participants in a study or increase the number of imaging timepoints collected per participant. A yearly high field image coupled with bi-monthly at-home lower field imaging, for example, would provide greater insight into early brain maturation and at lower cost than 2 semi-yearly conventional scans. Such at-home (or, in the case of older children, at-school) scanning could also significantly reduce participant burden, with corresponding benefits to retention.

An immediate challenge to including low field imaging into HBCD, however, is the reduced imaging quality and flexibility of systems like the Swoop compared to all-purpose higher field 3 T systems. For example, while image quality (signal-to-noise ratio, contrast, and spatial resolution) is impressive for the field strength, it falls short of 3 T. Further, while clinical high field systems can perform functional, spectroscopic, perfusion, diffusion, and structural scanning, the current range of imaging methods available at low field is limited to brain structure. It would be ill-advised, therefore, to image rural participants only with portable systems, and proximal urban participants with high field systems. However, this stratification could be avoided by instead viewing portable imaging to also increase imaging frequency across *all* participants. For example, if high field data were acquired where possible at 6, 12, and 24 months, low field data acquired at or near the participant’s home could be acquired at 3, 9, and 18 months, or in complement with EEG (see below). This would provide 2 complementary datasets, providing general trends of structural maturity, with a subset of specialized imaging measures.

### 2.3 Portable EEG

Most EEG monitoring systems currently available in clinical practice require wired attachments to a power supply, amplifiers, and base units, which dramatically limits their portability. Unlike MRI, EEG has a rich history of portable device development. Though most portable EEG systems had been developed for use with adults, or older children, there are now a variety of less expensive, portable, and reusable/disposable systems ranging from high density electrode nets to single channel sensors. Advancements in wearable, battery powered technologies and the development of adhesives specifically designed for placement and removal on fragile infant skin, now affords the opportunity for longitudinal remote investigations outside the traditional clinical environment. Moreover, recent efforts toward harmonization of EEG data across devices and preprocessing pipelines on standardized platforms offer options for increased complementary infant data acquisition and analysis (ref). As with lower field portable MRI, some lower density and disposable systems offer options for increased complementary data acquisition up to and including even daily continuous or pseudo continuous EEG measurements in a variety of settings.

**Fig. 3:**
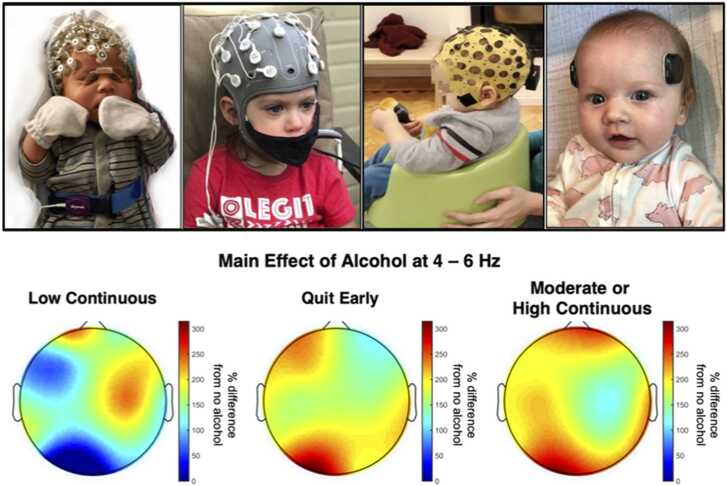
Top Panel (from left to right) 128-lead EGI net used to record EEG at birth in the nursery; 32-leads Enobio EEG system used at home to record EEG during an attention task in a toddler; infant wearing 8-leads Mentalab Explore system during tactile stimulus presentation; two Epilog devices (left and right forehead) used to record resting state EEG in a 5-month infant at home (right). Bottom Panel: Effect of alcohol at 4–6 Hz (theta) on newborn electroencephalography power representing the percent difference in power compared to reference group (no *in utero* alcohol exposure).

The Geodesic EEG systems (Magstim EGI, Inc) utilize high density nets ranging from 32 to 256 leads. The ensemble of dry electrodes evenly spaced over the entire scalp, cheeks, and the back of the neck, monitors brain activity with a high spatial and temporal resolution. The Geodesic system is less ‘portable’ than others as it currently requires nonportable additional pieces of equipment for data collection such as the dedicated amplifiers, and a computer Enobio EEG (Fig. 3) (Neuroelectrics, Inc) is a totally wireless EEG system suitable for mobile brain imaging and integration with other physiologic sensors. The device can store up to 20 h of continuous data on the internal memory. The Explore (Mentalab) is an inexpensive portable system (Mentalab) with a small profile, reusable caps and can collect simultaneous ECG.The Epilog device (Epitel, Inc.) is a miniature EEG device capable of recording high-fidelity EEG and ECG from single or multiple sensors. Epilog is inexpensive, disposable, and capable of recording continuous EEG data that is less susceptible to movement artifacts or antenna noise that frequently affect traditional wired EEG systems. The recorded EEG traces are stored on the device’s internal memory which can save up to 10 days of continuous data.

The analysis of EEG data offers the potential for quantification of the effects of adverse pregnancy exposures. The Safe Passage study investigated the effects of prenatal maternal drinking and smoking on infant brain activity EEG power at birth was computed from neonatal EEG traces recorded from ~ 1,700during natural sleep using 28-lead net. Fig. 3 shows the topological distribution of EEG power characterized by an increase in EEG power (4–6 Hz (theta)) in the several brain regions. Specifically, prenatal alcohol exposure resulted in increased low-frequency EEG power in a dose-dependent manner, such that infants with moderate or high continuous PAE had the most significant increase compared with infants with no PAE. Moreover, consistently for PAE and prenatal tobacco exposure, alteration of EEG power was asymmetrically distributed for the left and right hemispheres [REF].

While most of the the bove-described EEG systems require specialized training and it is impractical to be shipped to study participants, the development of user-friendly kits for remote data collection is undergoing. As an example, the small Epilog sensors can be used to collect EEG during either resting state or simple challenges (e.g., eyes open and eyes closed) in the home environment.

## 3 Neurocognitive assessment

Alongside neuroimaging, assessment of a child’s developing cognitive and behavioral functioning is a core component of HBCD. Depending on child age and the modalities of neuroimaging to be performed (i.e., during a daytime nap, nighttime sleep, or awake during the day), neurocognitive assessments may require a separate day of testing - necessitating additional travel or an overnight stay near the research facility.

Research and clinically administered neurocognitive batteries often include a mixture of performance-based and self- or parent-reported measures. In infants and toddlers, the Bayley Scales of Infant and Toddler Development (BSID) (Gauthier et al., 1999), Mullen Scales of Early Learning (MSEL) (Akshoomoff, 2006), and the Griffiths Scales of Child Development (Reyes et al., 2010) are amongst the most popular performance-based assessment tools. In older children, the Weschler scales, including the Preschool & Primary Scale of Intelligence (WPPSI) (Watkins and Beaujean, 2014), Intelligence Scale for Children (WISC) (Watkins, 2010), and the Adult Scale of Intelligence (WAIS) (Nelson et al., 2013, Canivez et al., 2017) being the de-facto standards for children as young as age 3 and older. Each of these scales are available in English and Spanish with appropriate population-normed references.

With respect to parent and self-reported measures, the Ages & Stages Questionnaire (ASQ) (Beswick, 2014), the Vineland Adaptive Behavior Scales (Perry and Factor, 1989), and the NIH Toolbox (Gershon et al., 2013) are widely used for general cognitive and intellectual development, as well as more specific functional domains. Alongside these tools, the Global Scales of Early Development and the NIH Infant and Toddler toolbox will, when completed, offer additional tablet-based assessments of children as young as 3 months across a range of languages.

As a result of the SARS-CoV-2 coronavirus and related Covid-19 pandemic, clinical services and research groups across the US hurried to develop remote assessment tools and protocols. For older children, the NIH Toolbox offers the potential for remote collection via videoconferencing. However, high speed internet and WiFi connectivity may not always be available. Approximately 30% of US households with an annual income less than $30,000 per year do not have access to high speed internet (Reddick et al., 2020).

In cases where direct observation or performance-based assessments cannot be performed i.e., for individuals who cannot accommodate multiple trips or prolonged time away from home, alternative approaches are needed. Mobile labs, such as those used to transport the portable MRI and/or EEG, with assessments performed in the RV or the participant’s home, offer an option or supplement to reliance on parent-reports observational measures. The latter, however, can be time consuming (lasting 2 + hrs for older children), and their general broad nature can be less sensitive to deficits in specific cognitive domains. To this end, free-viewing and task-based EEG methods in a mobile unit can also be used to assess language, motor skills, socioemotional reactivity and temperament, attention, executive functions, learning and memory – all functions believed to be potentially vulnerable to prenatal substance exposure.

A targeted and brief assessment battery comprising compatible in-person and on-line measures, such as the child Minnesota Executive Function Scale^185^ or NIH Toolbox^186^; developing language skills (NIH Toolbox^186^ and MacArthur Bates CDI^187^); socioemotional reactivity and temperament (IBQ-R^188^, ECBQ^189^ and the Lab-TAB^190^; attention and executive functioning (Gap/Overlap task^191^, MEFS^185^, and Go/No-Go ‘Zoo’ task^192^; learning and memory (visual paired comparison^193,194^, memory space/time tablet game^195^ and the NIH Toolbox picture sequence memory task^196^); and motor development (Vineland^197^ motor sub-domain) can be performed in less than 1 hr in all children regardless of assessment location.

## 4 Biospecimen collection

A thorough review of proposed HBCD biospecimen collection is provided by Croff et al. (2020) and so here we detail only specifics related to samples that can be readily collected remotely and considerations thereof, including sampling, storage, and shipping. Biospecimen collections, alone and in combination with neuroimaging, neurocognitive assessments, and validated questionnaires, are critical to establishing and disentangling prenatal substance exposures from the postnatal environment. The biospecimen working group highlighted essential and recommended biospecimens for biological mothers, fathers, and children across four domains of interest: a) substance use exposure; b) other environmental exposures; c) genomics and epigenomics; and d) other biological markers of neurodevelopment and putative moderators and mediators of developmental effects (Croff et al., 2020).

There are several distinct advantages to remote collection of biospecimens across, including the ability to capture additional samples from biological maternal and paternal sources, regardless of custody status. The consideration of remote biospecimen elements creates opportunities for collection of additional timepoints outside of those paired with in-person assessments.

While some collections (venous blood, for example) require specialized training to collect, the development of user-friendly and nearly painless collection and storage sets for saliva, hair, nail clippings, fecal, blood spots, and capillary blood; as well as at-home test kits for SARS-CoV-2, substance and alcohol use, has reduced the need for in-person collection of these samples. However, this does not mean collection kits can be simply mailed *en mass* to parents and families with the expectation that they can use and return quality samples. Care and thought must be given to packaging, presentation, and support. For example, well presented gift boxes with clearly labeled and separated kits, clear multi-lingual instructions (complemented by on-line instructional videos), and extra pens, labels, latex-free gloves, etc.

### 4.1 Assessment of substance use

Accurate measurement of prenatal exposures is a particularly important part of the upcoming HBCD study. Social desirability bias creates concerns around the validity of self-reported alcohol, tobacco, and other substance use during pregnancy. The biospecimen working group recommended a combination of biospecimens to capture recent use (e.g., urine drug screens) and use across a longer time period (i.e., fingernails for 3 – 6 months); the use of exhaled air in the postnatal period to assess recent tobacco and alcohol use/exposure; collection of breastmilk in the postnatal period; recommended cord tissue at delivery; and child urine and hair collection throughout childhood.

Maternal collection of fingernails is an essential element during pregnancy, and paternal collection is recommended during this time period. Fingernails lend themselves to self- and remote-collection; however, given the sensitivity of this biospecimen for the assessment of substance use during pregnancy, the final protocol should prioritize at least one in-person collection of this biospecimen.

Exhaled air breath collection devices for carbon monoxide (CO) and ethanol (EtOH) can be collected remotely, each with distinct exposure windows. Notably, EtOH breath tests are sensitive to error from alcohol remaining in the mouth after sipping an alcoholic drink, and gas from the digestive tract with high levels of EtOH. For these reasons, alternative remote collections may be preferred over breath-based systems. Notably, cotinine can be assessed via serum remotely collected using TASSO or TAP devices, or more affordably through dried blood spots, which have been validated for exposure in adults (Murphy et al., 2013) and infants (Spector et al., 2007). Continuous monitoring of alcohol use may also be accomplished by transdermal alcohol sensors, which have demonstrated utility among young adult women over periods as long as one month (Croff et al., 2020).

A breastmilk sample in the first month postpartum was requested for presence of opioids and other drugs. Breastmilk sampling will also inform infant nutrition and microbiome. Remotely collected breastmilk has been used to detect Δ9-THC (Bertrand et al., 2018) and other markers of cannabis use, organic contaminants (Tran et al., 2020), and SARS-CoV-2 (COVID-19) (Chambers et al., 2020). Because 19.2% of breastfed infants are supplemented with formula before they are 2 days old, remote sampling will help to capture this biospecimen before participants can be scheduled for in-person data collection.

Remote breastmilk collections can be paired with momsense or coroflow, both of which are smart devices to measure quantity of breastmilk consumed during a feeding, which can also act as incentives to parents and reassurance their child is receiving the appropriate nutrition.

For families who choose not to breastfeed, or who supplement breastmilk with formula, information on formula brand and approximate breastmilk / formula milk percentage in the child’s diet should be obtained.

### 4.2 Nutrition

Although nutrient deficiency is uncommon in the US, many Americans have inadequate intakes of several nutrients critical to neurodevelopment. These micronutrient inadequacies are critically important to measure during pregnancy. Inadequate nutrient intakes among women of childbearing age in the US have been identified for vitamin A, choline, B12, C, D, Calcium, and Iron. Various blood and serum collection devices including liquid blood collection devices (e.g., TAP, TASSO SST) and dried blood spots (with or without liquid chromatography, e.g., Whatman paper, TASSO-M20, ArrayIt) can allow for remote collection of blood during pregnancy in order to better understand the nutrients available throughout prenatal development. Deciduous teeth can also be remotely collected for the measurement of zinc and fatty acids (Camann et al., 2013) from the prenatal period through childhood. Breastmilk can also be collected to assess infant nutriture, with collection techniques described earlier.

While breastmilk, blood, and/or teeth may represent gold-standard approaches for nutrition intake, population health studies also employ food diaries and recalls, including the ASA-24 and similar (Subar et al., 2012, Jurgens et al., 2019). While available online, recall forms such as the ASA-24 can be time-consuming and exhaustive, leading to incomplete or missing data. To this end, new image-based approaches, which estimate nutritional intake from photographs of the meal or snack have been developed with validation on-going (Boushey et al., 2017). Such approaches may simplify the collection of nutrition data while providing robust and reliable estimates.

### 4.3 Neurotoxicant exposome

Known neurotoxicants of interest may create differential exposures by site. For example, former industrial hubs, including Rhode Island, Massachusetts, Pennsylvania, New York, Michigan, and Ohio have significant lead contamination in soil and ground water (Jurgens et al., 2019), which is known to impact brain development and cognitive outcomes (Yuan et al., 2006). Neurotoxicants, like heavy metals (e.g., lead, arsenic) and other environmental chemicals (e.g., phthalates, polychlorinated biphenyls (PBCs), and polybrominated diphenyl esters (PBDEs)) have broad windows of neurotoxicity, spanning prenatal and postnatal environments. Several remotely collected biospecimens of interest can be used to assess the NT exposome, including maternal prenatal serum samples, maternal prenatal urine, and offspring deciduous teeth, and breastmilk.

### 4.4 Genomics and epigenomics

Remote collection of saliva from maternal, paternal, and children can be used to link epigenetics changes in biological parents or other caregivers. Remote collection may enhance ability of biological parental participation in the research study, regardless of custody and parenting practices. Saliva collection is recommended by the HBCD biospecimen’s working group for generation of whole genome sequencing, and polygenic risk score generation.

### 4.5 Other biological markers of neurodevelopment

Microbiome. The gut-brain-microbiome axis plays a role in immune regulation and neurodevelopment. Working group recommendations included child stool samples. This work can be conducted remotely by caregivers in the home by swabbing diapers or toilet paper after (Costello et al., 2009, Herman et al., 2020) (Flores et al., 2014), which is particularly important because of changes in microbiome over time and by location.

During vaginal childbirth, which represents approximately 69.1% of births in the US, the infant is exposed to the vaginal microbiome. Along with other parts of the maternal microbiome, the vaginal microbiome colonizes the gut of the neonate. In animal models, the vaginal microbiome is sensitive to stressors, and has been tied to neurodevelopmental effects in offspring (Jasarevic et al., 2015). Patterns of the vaginal microbiome have been associated with preterm birth (Tabatabaei et al., 2019). Therefore, remotely collected samples of maternal vaginal microbiome after recruitment may serve as important variables of interest. Vaginal swabs are a highly-valid self-collected biomarker (Forney et al., 2010). Given the interest in microbiome colonization, swabs should be collected proximal to delivery; a strong case exists for frequent collection in the third trimester.

Depending on collection-type, microbiome samples can be collected and stored in commercial kits with preservatives, allowing them time to be couriered back to the research team. Care needs to be taken to ensure date and time of collection is noted, and that shipping time and conditions (e.g., summer in Arizona) are detailed.

## 5 Health and environmental monitoring

A child’s physiological health, home and built physical environment, and their surrounding socioeconomic, psychosocial, and socioemotional circumstances exert meaningful influences on their developing brain. However, deciphering the individual and cumulative contributions of these intertwined factors, in addition to those related to maternal antenatal health and substance use is challenging. This owes, in part, to the temporal variability of when these factors may be present, the brain’s changing sensitivity to them, and the brain’s relative developmental status. As an example, while maternal iron deficiency may be detrimental to the developing fetus throughout pregnancy, its effect may be most profound during the third trimester, when fetal iron stores are established for the early postnatal period, and iron sufficiency is critical for fetal hippocampal development and initial myelination of brainstem, cerebellar, and central deep white matter regions.

For many environmental and maternal and child health factors, however, the periods over which they exert their greatest influence are not known, nor their long-term effects. Thus, it may be desirable to collect information on nutrition status, substance use, air and water quality, neurotoxicant exposure, family income, neighborhood and schooling characteristics, and caregiving quality and caregiver-child interaction, and child physical activity, sleep, weight, body composition as often as possible. In the above section, we described methods that address some of these important aspects, such as blood spots, urine, hair, toe or fingernails, and breastmilk. In addition, the advancement of consumer wearables and ‘nearables’ have increasingly made routine and near continuous physiologic and environmental monitoring possible.

### 5.1 Physiology

**Fig. 4:**
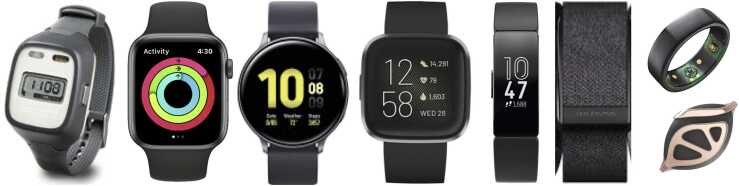
Example devices used for monitoring actigraphy and physiology, including the (left to right) Actigraph Spectrum, Apple Watch, Samsung watch, FitBit Versa, Fitbit Charge, Whoop band, and jewelry based trackers, (top to bottom) Oura ring and Bellabeat Leaf. All provide basic monitoring of physical activity and heart rate, with more advanced and expensive devices offering more in-depth physiological monitoring including ECG.

Apple Watch, Samsung Gear, Fitbit, Oura Ring, and other fitness trackers (Fig. 4) now provide continuous measures of physical activity level, energy expenditure (calories burned), heart rate and heart rate variability, blood oxygenation, body temperature, sleep health, as well as environmental sound exposure, glucose monitoring, and even electrocardiography (ECG). Beyond these built-in functions, connected wearables and nearables extend these capabilities to recording blood pressure, weight and body composition, spirometry and lung function, and air quality monitoring. Once the domain of expensive, bulky, and dedicated devices (e.g., actigraphy watches, Holter monitors), most commercial fitness monitors offer more expansive technologies in inexpensive and sleek form factors with integrated and curated data collection, presentation, and archival. Further, some of these devices, including those from Apple, Samsung, and Google, have adopted elements of open-source research frameworks (e.g., ResearchKit and ResearchStack for Apple and Android-based devices, respectively), that provide researchers with supported application programming interfaces (APIs) to access the underlying sensors and data streams, and the ability to develop custom study applications that can be used in controlled study environments, or made available to the general population. Studies have begun to evaluate the validity of these devices, with comparisons to clinical gold standards and methods revealing differences across devices. For example, Apple Watch has been found to have clinically acceptable measures of heart rate, heart rate variability, sleep/wake (Wallen et al., 2016; Walch et al., 2019), and incorporates an FDA-approved ECG method for atrial fibrillation detection (Inui et al., 2020); but overestimates measures of energy expenditure relative to indirect calorimetry. Other wearables, such as those from Fitbit, Samsung, and Oura, likewise show similar performance for heart rate and energy expenditure.

**Fig. 5:**
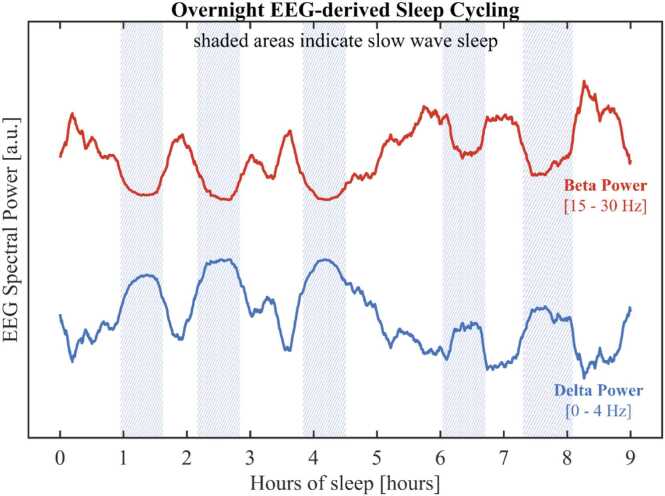
Overnight alternance of light and deep sleep cycles. EEG data were collected using a single lead wearable device worn by an adult subject on the forehead.

Regarding the characterization of sleep, wearable approaches, such as actigraphy, only capture sleep duration, efficiency, and latency, but can not provide information on sleep stage cycling. Newer devices can noninvasively acquire maternal EEG and ECG to characterize multidimensional measures of sleep in the home during pregnancy and in the months after delivery. As an example, EEG data acquired by a single lead wearable device (Epilog) can be used to derive overnight sleep cycling patterns as shown in Fig. 5. The quantification of EEG rhythmsin the in the delta and beta bands represent a valuable tool to monitor the cycling of deep and light sleep phases throughout the night. Moreover, such data could provide additional insights into maternal physical and mental health during pregnancy, including sleep, weight gain, overall activity, ecological momentary assessment of depression and anxiety. Coupled with smartphone, tablet, or web-based applications, such as Sprout Pregnancy or Ovia, these can provide a curated source of antenatal health, including medications, antenatal visits, and fetal growth measures. In children, continued sleep and physical activity monitors could provide valuable information into child growth, weight gain, and sleep health. For instance, one of the main limitations of previous sleep studies is that many have relied on subjective measures of sleep, such as parent-report questionnaires (e.g., Brief Infant Sleep Questionnaire), which do not fully characterize the complexity and multidimensional nature of sleep and are subject to reporting or recall bias. Primarily driven by the justified fear of sudden infant death (SIDS), there are increasing numbers of infant-centric devices that are particularly focused on sleep. For example, the clip-on MonBaby monitor records infant movement, position (including rolling over), and cardiorespiratory activity or devices like the Owlet Smart Sock or Gabi Baby Band, that use near infrared spectroscopy to measure heart rate and blood oxygen levels.

Using novel wearable devices, we can decrease subject burden by assessing the natural sleep environment. Importantly, sleep is a modifiable risk factor and practical tools to assess sleep health in the home environment will afford the opportunity to improve maternal health and promote health equity.

Furthermore, there is a lack of ecologically valid field studies (i.e., outside of the laboratory) and data are often collected at minimal time points, which may not accurately reflect possible changes across infancy-childhood.

Direct challenges to the adoption and widespread use of consumer wearables within the HBCD study, however, include: 1. Cost of hardware; 2. Participant applicability; 3. At-home infrastructure needs; and 4. Data integration and harmonization with lab-based collections.

When paired with a smartphone, for example, even the lowest cost Apple Watch and refurbished unlocked iPhone combination is a significant investment of hundreds of dollars per study participant – even before additional specialized ‘smart’ devices are added, e.g., blood glucose and blood pressure sensors While wearable throughout pregnancy by mothers, and by older childhood (~5 years of age and older), most wrist or finger-based fitness and health monitors are not size-appropriate or validated in infants and young children. The reliance of many wearables on WiFi connectivity and short battery life are important considerations. As mentioned above, fast and stable internet connectivity cannot always be assumed, particularly amongst the more rural and lower income settings these devices would be most useable. The need to recharge devices 3 or more times per week can lead to missed data collection. To address these challenges, the newest wearable technologies offer options to collect important physiologic metrics at a lower price point, across the targeted HBCD age spectrum, with longer battery life and viable connectivity protocols (e.g., Bluetooth). For example devices like the Fitbit Zip or Spire Health Tag last several months allowing the device to be handed out at one study visit and collected, synced, and returned at the following with minimal interference to the participant’s routine.

Cumulatively, therefore, numerous options exist to measure basic physical activity and general cardiovascular health. However, differences in sensor performance, measurement techniques, and calculation algorithms between devices (including those from the same vendor) can challenge the integration of measures across them and implies the need for a consistent and homogeneous approach in HBCD.

### 5.2 Physical environment (air and chemical exposures)

Like physical activity, general concern of air quality and pollution on child health (Buka et al., 2006, Koranteng et al., 2007) has led to the proliferation of personal wearable and nearable air quality monitors to track carbon monoxide, carbon dioxide, and pollutants such as dust, pollen soot, and mold, as well as volatile organic compounds (VOCs), alongside temperature and humidity. In particular, most higher quality devices focus on measuring inhalable particles with diameters up to 2.5micrometers (PM2.5), which have been associated with reduced child lung health, obesity, cognitive impairments, and academic difficulties (Buka et al., 2006, Koranteng et al., 2007, Friedrich, 2018, Payne-Sturges et al., 2019, Seo et al., 2020).

**Fig. 6:**
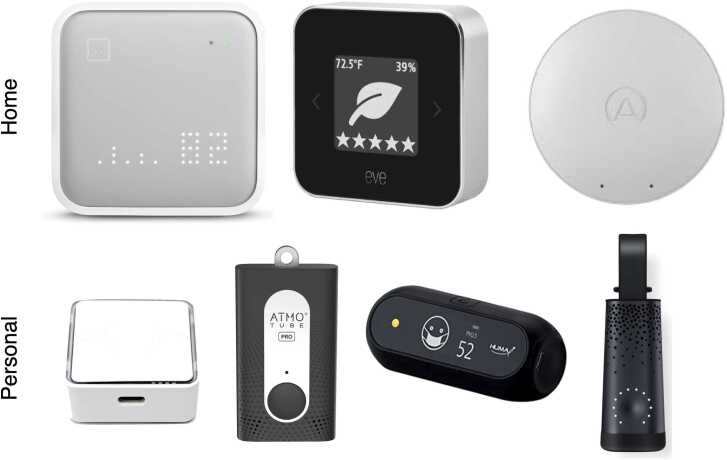
At-home and personal air quality monitors including (top, left to right): AWAIR, Eve, and Airthings room monitors; and (bottom, left ti right): Pico, Atmotube, Huma, and Flow personal wearable monitors.

At home monitors (Fig. 6), such as those from AWAIR, Eve, Airthings, IQAir, and others offer a range of devices from $49 to $499 that provide a basic set of temperature, humidity, carbon dioxide, and VOC measures, that can be connected to a smartphone either via WiFi or Bluetooth. These can be placed in main rooms of the house, including the bedrooms and nursery, to gauge overall trends in air quality throughout the house.

For portable monitoring, the Pico, Airbeam, Flow, and Atmotube are wearable devices that are worn around the neck or attached to a waistband. They provide more personalized measurement, which may be valuable for working parents and school-aged children who spend significant time outside the house.

Less technologically savvy, silicone wristbands can inform not only on aspects of air quality, but also numerous chemical exposures (Dixon et al., 2019). These passive ‘devices’ are cheap, simple to wear by every member of the household (including four-legged members), and can be stored for later analysis (Dixon et al., 2018, Doherty et al., 2020). The principle drawback to these, however, is the cost of analysis, which can range from several hundred to thousands of dollars each.

### 5.3 Light and media exposure

Smartphones, tablets, and laptops have become go-to entertainment and content-consumption devices for parents and their children, with toy makers even designing infant-friendly protectors (e.g., Fisher-Price Laugh & Lean iPhone and iPad Case). However, in addition to taking away time that could otherwise be used for more interactive or exploratory play, these devices can also emit blue light that interrupts sleep patterns and circadian rhythms, impacts memory and general cognitive function, and may impact overall child health (including obesity) and eyesight development (Read et al., 2015, O'Hagan et al., 2016, Pattinson et al., 2016, Chaopu et al., 2018, Tonetti and Natale, 2019).

Makers of Digital devices (principally Apple, Samsung, and Google) now include screen time monitors and light shifting (e.g., NightShift) technologies within their mobile OSes that provide tracking information on total time spent on the device curated into general categories (gaming, social media, reading and reference, etc.), as well as more granular information including time spent in individual apps.

Beyond media exposure, however, light exposure throughout the day is also associated with overall health. Activity monitors, such as the Philips Actigraph Spectrum, the CamNtech MotionWatch, and the Condor ActTrust offer not only activity monitoring but also include multiple wavelength ambient light exposure sensors. These devices, however, are made for adults and are not form fitted for infants or toddlers, and can be cumbersome.

### 5.4 Caregiver interaction

Optimal brain development requires secure and trusting relationships with knowledgeable caregivers who are responsive to the infant’s needs and interests. Fundamental processes of neurodevelopment, including myelination and synaptogenesis, for example, are stimulated by external cues and experiences like maternal interaction, and physical skin-to-skin “kangaroo” care, touch, and warmth. Caregiver responsiveness, interaction, and skin-to-skin care are further associated with general health metrics, including immune function, weight gain, and prolonged breastfeeding exclusivity, which themselves also promote improved brain development.

Quantification of caregiver interaction, however, is often laborious and time consuming and, when performed under novel laboratory conditions can result in biased findings. Throughout early development caregiver-child interaction, including language exposure (infant-directed speech, conversational interaction), has also been shown to be an important precursor and predictor of later school readiness and academic success (Duncan et al., 2007, Forget-Dubois et al., 2009). Past work has crystalized the ’30 million word gap’ - an estimate of the difference in the number of words children from higher income families are exposed to compared to those from lower incomes. Differences in early language exposure and interaction (specifically, conversational turns, which reflect a back-and-forth interaction between the child and their caregiver) have been associated with increased child language comprehension and school readiness, as well as increased functional and structural brain connectivity in infants as young as 6 months (Romeo et al., 2018, King et al., 2020) and functional word processing in order children (Romeo et al., 2018).

The current gold standard wearable for at-home language assessment is the Language ENvironment Analysis (LENA) device, which is about the size of a deck of cards and records the child’s environment for up to 8 continuous hours. These recordings are then analyzed and broken down into various metrics, including the total number of adult words the child hears, the number of vocalizations the child makes, and the number of conversational turns (child response to adult word and vice versa). Other metrics, including the amount of time a television is present, are also provided. Conventionally, at least 16 h (approximately 2 days) of recording are preferred, which can be a challenge with the device recording limit and the need to physically transfer data to a laptop. A recent introduction has added a cloud-based option for storage and analysis, however, this still requires at least 2 devices to be provided to each child. A further concern is the recorded audio file creates a potential confidentiality and security risk.

Newer technologies, ranging from ‘smart’ speakers (e.g., ECHO, HomePod, Nest, Sonos, and others may provide a technical platform for recording and analyzing adult and infant directed speech remotely across multiple languages, and throughout the home, without need for a wearable device. Alternatively, the low cost of digital sensors, memory storage, and increasing language and motion processing capabilities of smart devices make it easy to imagine wearables that combine activity, light, physiology, and language environment monitors into a coin-sized device.

## 6 Discussion

The proliferation of low-cost consumer, and ‘prosumer’ electronic sensors, together with active open-source and manufacturer-supporter developer communities, have led to a proliferation of wearables and ‘nearables’ that offer the potential to replace established high-cost standards. Many of the devices illustrated above, including the larger imaging systems, have only become available over the last 12–18 months, and offer compelling new approaches to collecting multiple forms of data reliably. However, beyond the specific cost, infrastructure/connectivity, and applicability for infants, toddlers, and young children; additional practical challenges and hurdles remain. The first of these is device overload. While making use of existing and current devices is appealing from an availability, warrantee, and service perspective, many devices offer focused or narrow ranges of data. Thus, multiple devices, often requiring the same wrist real estate are needed to obtain the full spectrum of desired data (i.e., an Apple Watch for physiology; an actigraphy watch for sleep and light; Atmotube for environmental air quality; etc.), in addition to the participant’s own devices. Thus, custom-built wearables that merge many functionalities may be preferable, but may not have the same degree of design esthetic, battery life, or user-friendliness that participants may be used to and expect.

For many commercial devices, access to raw data and algorithm specifics is tightly regulated. Some commercial partners may require legal research and non-disclosure agreements, which may impair sharing of data and open publishing; or only processed and aggregated data may be made available, limiting the types and extent of follow-on analysis that may be performed. While smaller vendors may be more willing to work with individual researchers to make data available, or to help validate algorithms and supplied metrics against established gold standard devices or questionnaires, this is not a universal truth. These companies may not have the resources to build or support developer APIs for their devices.

Device and data harmonization and integration will be a continuing challenge given the fast-paced nature of change in consumer wearables. Sensor upgrades, algorithm updates t, and other modifications across device generations from the same vendor, or between devices from different vendors, make it difficult to directly combine and integrate data without performing rigorous comparisons (particularly so if access to the raw data and/or underlying algorithms is not possible). This may argue for standardizing on a device and platform at study outset. However, devices are likely to change substantially over the planned 10-year study duration of HBCD. For example, the first Fitbit was only released in 2009, the first iPad in 2012, and the first Apple Watch in 2015, and all of which would be considered antiquated, slow, and of limited functionally compared to current iterations. Thus, standardizing now will likely mean limiting access to future functionality or advancement.

As recent headlines of banking and hospital data breaches have made clear, connected devices and the ‘internet of things’ are increasingly attractive targets for cyber criminals. As devices become more capable, interconnected, and filled with more personalized information, the need to secure them is ever-present. Location data, health metrics, financial information, and recorded conversations and interactions are sensitive information that need safeguarding and protection beyond those outlined in the Health Insurance Portability and Accountability Act (HIPAA) (Colorafi and Bailey, 2016, Kayaalp, 2018) and General Data Protection Regulation (GDPR) (Bovenberg and Almeida, 2019, Clarke et al., 2019) regulations. Some possible avenues to address these concerns include on-device analysis (for example, performing language environment analysis on the device and storing only the derived measures rather than retaining the full audio recording); on-device anonymization and data encryption; forgoing wireless or Bluetooth connectivity and opting for connected data transfer from the device to the intended data repository; and secure enclaves within the device (e.g., Apple T2 chip and Secure Enclave) (Abu-Tair et al., 2020, Ilokah and Eklund, 2020). Neuroimaging data, in particular MRI of the brain and head, offers additional and unique challenges since surface rending techniques can permit potential facial recognition and subject identification (Prior et al., 2009). Skull stripping and other masking techniques be applied immediately following acquisition (Budin et al., 2008) may help reduce this possibility. In addition, the use of blockchain to track and monitor data access, modifications, and processing updates in a decentralized manner can further ensure trusted analysis while providing noneditable data provenance ledgers (Huang et al., 2020).

### 6.1 Participant-centered collection

While it is critical that captured data and measures be reliable, consistent, validated, and secure, the sine qua non that the data be measured in the first place. While each individual collection kit may not be burdensome or confusing on their own, multiple sample collection kits and devices with different timings and instructions can quickly become onerous and confusing. Further, as study participants increasingly own their own set of devices it becomes more and more difficult to encourage them to adopt a different study-specific device. Applying HCD principles requires looking at the complete study through the eyes of the participating family and child and making the process as transparent and minimally invasive as possible. This challenge begins the moment the participant receives the package of devices, kits, and tubes; and ends when they are received back by the study team.

It is critical to give thought and attention to how devices are sent, how they are packaged, and what instructions are included. Packaging and cadence of delivery of remote collection materials is a key consideration. Indeed, like commercial packaging, remote device packaging serves as a mechanism for highlighting content, promoting engagement and enhancing communication opportunities with participants. Principles of packaging design require that a great deal is known about the end user, and their needs surrounding supported use of the products (e.g., packaging hot spots already paired to wearable devices for those without reliable home WiFi, or pre-loaded software to eliminate complicated, finicky or lengthy set-up).

The goal of packaging is to encourage the participant’s continued interaction with the research product – and return of data. Therefore, it is important that the remote collection packaging clearly communicate without additional tools. Packaging is known to influence the uptake and use of medicines and the perceived flavor of foods; it is critical to design the remote collection package in a way that sequentially introduces participants to layers of data collection devices. Human-centered design must integrate clear directions, and colors that engage and encourage participants to interact and engage with research products and remote collection inside. For example, microbiome collection kit should be packed separately from the genetic, urine, or other kits in their own color that can be consistently identified. Kits for different daily collections could extend this further by using different shades with clear identifying labels (e.g., Day 1, Day 2, etc.).

Taken together, appropriate packaging and a user-friendly and engaging remote collection experience serves as an opportunity to enhance the brand and mission of the HBCD study.

## 7 Conclusion

While the importance of human-centered design is often overlooked in health research, we contend that remote data collection represents an embrace of its practices - placing the participant and not the laboratory setting, at the center of the research experience, and designing metrics, measures, and collection techniques around them and their lives. Within the context of the HBCD study, success requires the participation of families from all walks of life, across racial, ethnic, demographic, and socioeconomic spectra, and geographic division. From both a participant recruitment standpoint and the need to acquire detailed information across multiple domains of maternal and child health, exposures, and environmental conditions, remote data collection must be a central feature of the HBCD study design. Here we have highlighted the opportunities and addressed the challenges likely to be encountered as the final study is developed. However, there can be little doubt that HBCD will be a defining study in advancing the use of remote data capture and embracing the innovation of portable imaging, health wearables, and environmental nearables.

## Funding

SCD UG3OD023313, R34DA050284, OPP-005774, OPP-006627 JMC R34DA050343.

## Data statement

No novel data was acquired or included as part of this review article.

## Declaration of interest

SCD receives grant funding and consulting salary from Nestle SA, Wyeth Nutrition, and Mead-Johnson Nutrition. Neither of these conflict with the manuscript.

No other authors reports a significant conflict of interest.
